# Polymerizable Ionic Liquid-Based Gel Polymer Electrolytes Enabled by High-Energy Electron Beam for High-Performance Lithium-Ion Batteries

**DOI:** 10.3390/gels10120798

**Published:** 2024-12-06

**Authors:** Wookil Chae, Taeshik Earmme

**Affiliations:** Department of Chemical Engineering, Hongik University, Seoul 04066, Republic of Korea

**Keywords:** polymer electrolytes, in situ polymerization, lithium-ion batteries, electron beam, ionic liquids

## Abstract

Polymerizable ionic liquid-based gel polymer electrolytes (PIL-GPEs) were developed for the first time using high-energy electron beam irradiation for high-performance lithium-ion batteries (LIBs). By incorporating an imidazolium-based ionic liquid (PIL) into the polymer network, PIL-GPEs achieved high ionic conductivity (1.90 mS cm^−1^ at 25 °C), a lithium transference number of 0.62, and an electrochemical stability exceeding 5 V. E-beam irradiation enabled rapid polymer network formation within a metal-cased battery structure, eliminating the need for initiators and improving the process efficiency. In the NCM811/PIL-GPE/Li cells, PIL-GPE (8:2) delivered an initial discharge capacity of 198.8 mAh g^−1^ with 82% retention at 100 cycles, demonstrating enhanced thermal stability and cycling performance compared to traditional GPEs. The demonstrated PIL-GPEs demonstrate strong potential for high-stability, high-performance LIB applications.

## 1. Introduction

The commercialization of lithium-ion batteries (LIBs) by Sony in 1991 revolutionized portable electronics, showing a significant surge in LIB research. This attention has led to advancements in various aspects of LIB performance, yet safety concerns remain unresolved [1,2,3,4]. These safety issues are primarily attributed to the liquid electrolytes (LEs) that use organic solvents, which has intensified interest in solid-state electrolytes (SSEs) as a safer alternative to LEs [5]. Despite oxide, sulfide, and polymer-based SSEs having received a lot of attention, oxide-based SSEs suffer from interfacial problems with the electrode, sulfide-based SSEs are chemically unstable, and polymer-based SSEs suffer from low ionic conductivity at room temperature [6,7,8]. Addressing these issues requires substantial ongoing research.

Gel polymer electrolytes (GPEs) consist of liquid electrolytes (LEs) entrapped within a polymer matrix, forming a structure in which both phases are continuously connected and exhibit properties between those of LEs and solid polymer electrolytes (SPEs). GPEs are considered a viable alternative to LEs due to their excellent ionic conductivity, processability, and high stability, particularly in terms of electrolyte leakage and flammability [9]. The ionic conduction mechanism in GPEs generally aligns with that of LEs; however, the ionic conductivity is largely influenced by interactions between the polymer and the charge carriers [10].

Recently, in situ polymerization has gained considerable attention as a promising approach for preparing polymer electrolytes (PEs) [11,12]. Traditional ex situ methods used to fabricate conventional PEs, such as solution casting and electrospinning, face the challenge of achieving perfect interfacial contact between the electrode and electrolyte, leading to limited ion transport across certain areas during LIB operations [13,14]. In situ polymerization involves injecting a precursor in its initial liquid state onto an electrode, followed by physical or chemical crosslinking to form a polymer network. The liquid electrolytes (LEs) become entrapped within this polymer network to form GPEs, which can exhibit excellent interfacial contact with the electrode.

A typical method used in in situ polymerization fabricates GPEs through the thermal or UV treatment of a liquid precursor composed of polymerizable monomers, such as those with vinyl functional groups [15,16]. However, thermal initiation can take anywhere from several minutes to hours, delaying the overall fabrication time, while UV treatment cannot penetrate the metal casing of fully assembled LIBs to initiate polymerization. As a result, all UV exposure must occur with the unassembled battery cell in an exposed condition, which is challenging to control. These limitations make thermal and UV treatments challenging for practical battery manufacturing.

Electron beam (E-beam) irradiation, generated by an electron accelerator, offers an alternative by facilitating the polymerization and crosslinking of polymers at room temperature without initiators [17]. A 10 MeV E-beam offers excellent processability due to its high penetrating power, capable of passing through several centimeters of water, and its high dose rate of 3–15 kGy s^−1^ [18,19,20,21,22]. In situ polymerization with E-beam irradiation, leveraging these characteristics, enables rapid polymerization through metal casings without initiators, showing a strong potential for application in the manufacturing process of polymer-based LIBs.

Room temperature ionic liquids (RTILs) are materials that remain in a liquid state at room temperature due to the low interactions between the ions, resulting from the size of the asymmetry of the cations and anions [23]. Typical RTILs include the imidazolium, pyrrolidinium, and ammonium series, which offer properties such as high thermal stability, non-flammability, and broad electrochemical stability [24,25,26]. Additionally, when incorporated into LIB electrolytes, RTILs can enhance both ionic conductivity and mechanical properties, making them highly attractive materials for LIB applications [27].

In this study, we introduced an imidazolium-based polymerizable ionic liquid (PIL) to create a polymer network for GPEs. In situ polymerization with E-beam irradiation was conducted to fabricate the GPEs, resulting in a polymer network formed within a very short time without the need for initiators, effectively entrapping the liquid electrolyte inside the polymeric matrix. The PIL-based polymer network facilitated facile Li+ movement within the GPEs, enabling the developed PIL-GPE (8:2) to achieve an ionic conductivity of up to 1.90 mS cm^−1^. PIL-GPE (8:2) demonstrated high lithium transfer number of 0.62 and electrochemical stability up to 5.3 V by suppressing the anion decomposition, attributed to the electrostatic attraction between the imidazolium cationic group of PIL and the anion. In the LiNi_0·8_Co_0·1_Mn_0·1_O_2_ (NCM 811)/PIL-GPE (8:2)/Li cell, PIL-GPE (8:2) demonstrated an initial discharge capacity of 198.8 mAh g^−1^ and retained 82% of its initial capacity after 100 cycles at 0.3 C-rate.

## 2. Results and Discussion

GPEs were prepared using a 10 MeV high-energy E-beam irradiation. During irradiation, accelerated electrons with high penetrating power passed through the stainless-steel coin cell exterior and interacted with the monomer. This process generated radicals without the need for initiators, forming a 3D polymer network through the crosslinking of trimethylolpropane ethoxylate triacrylate (ETPTA) and 1-[2-acryloyloxyethyl]-3-butylimidazolium bis(trifluoromethanesulfonyl)imide ([AEBI][TFSI]) monomers, both of which contained acrylate groups. The [AEBI][TFSI] monomers were synthesized following the previously reported method [28], and the detailed synthetic scheme is shown in the Methods section. The obtained polymer network through the polymerization of ETPTA and [AEBI][TFSI] could effectively encapsulate liquid-state plastic crystal electrolytes (PCE), composed of succinonitrile (SN) and lithium salt (LiTFSI), thereby creating polymeric gel (Figure S1).

**Figure 1:**
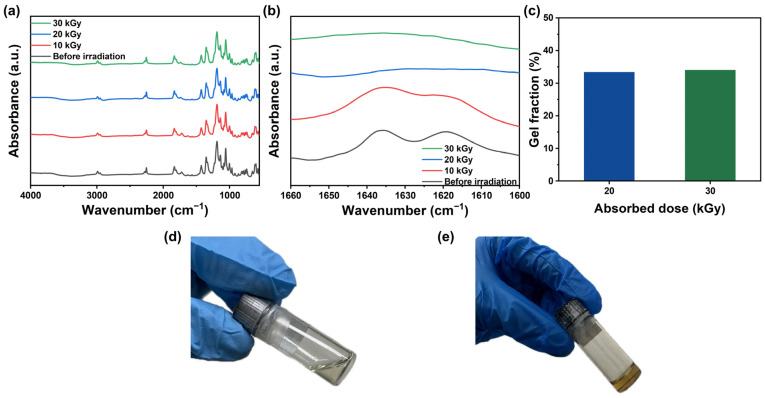
(**a**) FT-IR spectra of the PIL-GPE (8:2) before and after E-beam irradiation; (**b**) FT-IR spectra representing the C=C band of the acrylate monomers of the PIL-GPE (8:2); (**c**) gel fraction of the PIL-GPE (8:2) at 20 and 30 kGy; (**d**) liquid state precursor solution before E-beam irradiation; and (**e**) gel state PIL-GPE (8:2) after the irradiation.

Fourier-transform infrared spectroscopy (FT-IR) analysis was conducted to confirm the formation of polymer networks by polymerizing the polymerizable ionic liquids (PILs) using E-beam irradiation. The FT-IR spectra for the highest proportion of PIL in the polymer network, PIL = 8:2 (PIL-GPE (8:2)), revealed a peak corresponding to the C=C bond at 1620–1640 cm^−1^ before E-beam irradiation (Figure 1a,b). Upon E-beam irradiation at an absorbed dose of 10 kGy, the intensity of this peak gradually decreased, indicating that polymerization occurred and thus that the crosslinked monomers were forming a polymer network. The peak disappeared entirely at an absorbed dose of 20 kGy, with the spectra at 30 kGy matching that of 20 kGy. This confirmed that a fully developed polymer network was obtained at an absorbed dose of 20 kGy, effectively entrapping the liquid-state PCE within the network.

Furthermore, no changes were observed in the peaks across the other wavenumber regions, except for a variation in the intensity of the peak corresponding to the C=C band (Figure 1a), even after irradiation with the highest absorbed dose of 30 kGy. This suggests that PIL-GPE (8:2) is stable under E-beam irradiation, as no degradation was detected in the PIL-GPE (8:2).

The gel fraction of the PIL-GPE (8:2) was measured, which represents the proportion of the polymer network in GPEs. A higher gel fraction indicates greater crosslinking density which could contribute to enhanced mechanical robustness [29]. As shown in Figure 1c, the PIL-GPE (8:2) irradiated with 20 and 30 kGy E-beam doses, exhibiting similar gel fraction values of 33.4% and 34.0%, respectively, suggesting that additional crosslinking reactions did not occur at higher dose value of 30 kGy, and thus that 20 kGy of dosage was sufficient to form polymeric gel network structure. A 20 kGy absorbed dose, corresponding to 18 s of electron beam irradiation, prompted the monomers in the precursor to react and form a crosslinked polymer network.

As illustrated in Figure 1d,e, the precursor with SN, LiTFSI, [AEBI][TFSI], and ETPTA, was in a liquid state prior to E-beam irradiation. After exposure to an E-beam at an absorbed dose of 20 kGy, the precursor transformed into a gel through polymerization. This transformation suggests that safety concerns, such as LE vaporization at high temperature or leakage, can be effectively mitigated by the formed GPE.

**Figure 2:**
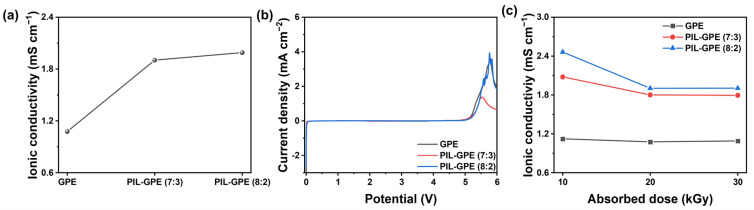
Electrochemical characteristics as a function of the proportion of PIL in the polymer matrix: (**a**) ionic conductivity, (**b**) electrochemical stability, and (**c**) ionic conductivity of GPEs at various absorbed doses.

Ionic conductivity is a critical parameter in the performance of LIBs, as high ionic conductivity enables rapid ion transport between electrodes, directly impacting the efficiency and charge/discharge rate capability of batteries. Typically, LEs in LIBs exhibit ionic conductivities ranging from 10^−3^ to 10^−2^ S cm^−1^ [30,31]. To create GPEs as viable alternatives to LEs in LIB applications, they must achieve an ionic conductivity of approximately 10^−3^ S cm^−1^ or higher [11]. As shown in Figure 2a, the ionic conductivity was measured as a function of the PIL proportion in the polymer network.

The GPE formed solely from ETPTA as the polymer network demonstrated an ionic conductivity of 1.08 mS cm^−1^ at 25 °C. By incorporating PIL into the network, the ionic conductivity was improved. The polymer network with a PIL at a ratio of 7:3 (PIL-GPE (7:3)) showed an increased ionic conductivity of 1.80 mS cm^−1^ and, using a PIL-GPE (8:2) with a higher PIL proportion, exhibited the highest ionic conductivity of 1.90 mS cm^−1^. We also prepared PIL-GPE with a ratio of 9:1. However, E-beam polymerization only increased the viscosity of the precursor without achieving a gel state. Therefore, we determined that 8:2 is the maximum ratio of PILs that can be used effectively. The observed increase in the ionic conductivity confirms that the incorporated PIL content in the polymer network effectively enhanced the ionic conductivity. This enhancement in ionic conductivity is due to the relatively large cationic PILs, which create additional local free volume within the polymer network. This expanded space increases the liquid-like environment that facilitates Li^+^ ion mobility, thereby improving ion transport [32]. Additionally, the electrostatic repulsion between the cationic groups in the ionic liquid and the Li^+^ ions reduces the binding force, enabling Li^+^ ions to move more freely and efficiently within the GPE [33].

The electrochemical stability of GPEs was evaluated by systematically increasing the proportion of PIL in the polymer network. As shown in Figure 2b, the oxidation stability of the GPEs improved with a higher proportion of PIL. Specifically, the GPE without PIL displayed electrochemical decomposition at 5.1 V, while the PIL-GPE (7:3) decomposed at 5.2 V, and the PIL-GPE (8:2) exhibited even higher decomposition behavior at 5.3 V. This trend demonstrates that incorporating more PIL enhances the resistance of the GPE to oxidize at higher voltages. All three of the electrolytes demonstrated high electrochemical stability above 5 V, even after 2–3 repeated measurements, highlighting their suitability for applications with high-voltage cathodes and extended operating voltage ranges. The increased electrochemical stability with higher PIL content is likely due to the robust ionic network provided by the PILs, which contributes to a more electrochemically stable electrolyte structure and mitigates the oxidative breakdown at high voltages. This stability is essential for high-voltage applications in lithium-ion batteries, where oxidative stability limits can directly impact battery lifespan and safety. Considering both ionic conductivity and electrochemical stability, PIL-GPE (8:2) was selected as the optimal polymer network composition, balancing these properties effectively for potential use in high-performance, high-voltage LIBs.

Figure 2c illustrates the changes in the ionic conductivity of GPEs when irradiated with E-beam doses ranging from 10 to 30 kGy. The ionic conductivity of the standard GPE remained relatively constant, showing values of 1.12, 1.07, and 1.09 mS cm^−1^ at doses of 10, 20, and 30 kGy, respectively. This consistency is likely due to the dense crosslinked polymer network formed even at the initial 10 kGy dose, as ETPTA has three reactive sites that can rapidly create a robust network. Since the network structure was substantially formed at 10 kGy, further irradiation did not significantly alter the polymer structure, resulting in minimal changes to the ionic conductivity.

In contrast, the PIL-GPE (7:3) showed ionic conductivities of 2.08, 1.80, and 1.79 mS cm^−1^ at 10, 20, and 30 kGy, respectively, while the PIL-GPE (8:2) displayed the highest ionic conductivities of 2.46, 1.90, and 1.90 mS cm^−1^ at the same respective doses. The high ionic conductivities of the PIL-GPE (7:3) and the PIL-GPE (8:2) at 10 kGy exceeding 2.0 mS cm^−1^ can be explained by the incomplete formation of the polymeric network at this lower dose, as confirmed by the FT-IR spectra. At this stage, the network remained partially liquid, allowing the Li^+^ ions to show greater mobility than in a fully crosslinked gel, resulting in higher ionic conductivity. Once the E-beam dose reached 20 kGy, the ionic conductivity decreased slightly in both the PIL-GPE (7:3) and the PIL-GPE (8:2) as the polymer network became fully crosslinked, reducing the liquid state and thus the Li^+^ mobility. Furthermore, the ionic conductivities of thet PIL-GPE (7:3) and he PIL-GPE (8:2) remained similar from 20 to 30 kGy, as the polymer network had fully developed by 20 kGy, with no significant increase in the crosslink density at the higher doses, as indicated by the gel fraction analysis. This suggests that additional irradiation beyond 20 kGy does not affect the polymer network structure or ionic conductivity.

We determined the lithium transference numbers (*t_Li+_*) of the control GPE without PIL and PIL-GPE (8:2) with the optimized ratio and absorbed dose of the PIL. The lithium transference number *t_Li+_*, indicated the fraction of the total current carried by lithium ions and was calculated using the Bruce–Vincent method. To obtain *t_Li+_*, electrochemical impedance spectroscopy (EIS) measurements were conducted before and after polarization in a Li/Li symmetric cell, combined with chronoamperometry to monitor the current variation over time.

**Figure 3:**
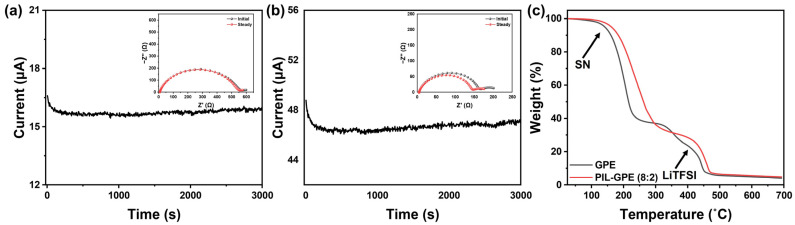
Lithium transference numbers of (**a**) GPE and (**b**) PIL-GPE (8:2); and (**c**) TGA thermogram of GPE and PIL-GPE (8:2).

As shown in Figure 3a,b, GPE and PIL-GPE (8:2) exhibited *t_Li+_* values of 0.50 and 0.62, respectively. The higher *t_Li+_* value of PIL-GPE (8:2) compared to the GPE demonstrates improved lithium-ion transport. This enhancement is attributed to the immobilization of the TFSI^−^ anions by the cationic groups in the ionic liquid, which engaged in electrostatic attraction with the TFSI^−^. This interaction reduced the mobility of the TFSI^−^ and thus effectively increased the lithium transference number by allowing a greater proportion of the current to be carried by the Li^+^ ions [33]. Based on the calculated ionic conductivity from the lithium ions ($\sigma_{tLi+}=\sigma \times t_{Li+}$), the GPE showed a value of 0.535 mS cm^−1^, while the PIL-GPE (8:2) had a higher value of 1.18 mS cm^−1^. This result indicates that PIL-GPE (8:2) can possess superior electrolyte performance at high charge/discharge rates, enhancing its suitability for high-performance applications [34]. The improvement in the electrochemical stability with the incorporation of PIL into the polymer network can also be attributed to the electrostatic attraction between the cationic groups of the ionic liquid and the TFSI^−^ anions. Electrolyte instability at high voltages generally results from anion decomposition [35]. As previously noted, the cationic groups in the polymer network immobilized the TFSI^−^ anions, effectively reducing their mobility and suppressing anionic decomposition, which in turn enhanced the stability of the GPEs under high-voltage conditions. This stabilization mechanism makes PIL-GPE (8:2) a promising candidate for applications in high-voltage lithium-ion batteries.

The thermal stability of the GPE and the PIL-GPE (8:2) was evaluated using thermogravimetric analysis (TGA). The decomposition onset temperature was determined at which a 5% weight loss from the original mass occurred. As illustrated in Figure 3c, the thermal decomposition temperatures for the GPE and the PIL-GPE (8:2) were found to be 142 °C and 162 °C, respectively.

The higher decomposition temperature of the PIL-GPE (8:2) indicates improved thermal stability compared to GPE, likely due to the enhanced structural stability provided by the PIL component. The initial rapid weight loss observed in both the GPE and the PIL-GPE (8:2) around 130 °C is primarily attributed to the evaporation of succinonitrile (SN), a volatile component in the electrolyte. The presence of PIL in the polymer network seems to contribute to the overall thermal stability, making the PIL-GPE (8:2) more resistant to decomposition at elevated temperatures. This enhanced thermal robustness is advantageous for lithium-ion battery applications, where higher thermal stability can contribute to greater safety and extended operational lifespans under high-temperature conditions [36].

The GPE demonstrated decomposition of the polymer network starting at approximately 330 °C, followed by the decomposition of the LiTFSI around 400 °C. In contrast, the PIL-GPE (8:2) exhibited polymer network decomposition only above 400 °C. This result confirms that the PIL-based polymer network offers superior thermal stability, highlighting its potential for reliable operation of LIBs under high-temperature conditions.

**Figure 4:**
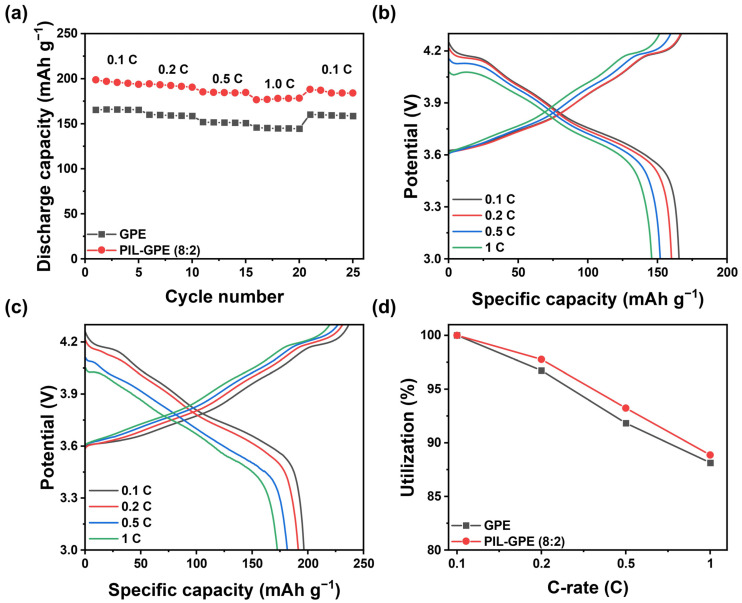
(**a**) Discharge capacities of GPE and PIL-GPE (8:2) using NCM 811 cathode and Li anode at various C-rates; charge/discharge profile of (**b**) GPE, (**c**) PIL-GPE (8:2); and (**d**) capacity utilization ratios of GPE and PIL-GPE (8:2) at various C-rates.

CR2032 coin-type cells with an NCM 811/GPEs/Li configuration were fabricated to evaluate the performance of the PIL-GPE (8:2). The discharge capacity of the PIL-GPE (8:2) was tested at various C-rates, ranging from 0.1 C to 1 C at 25 °C (Figure 4a and Table 1). The GPE containing only ETPTA as the polymer network displayed discharge capacities of 165.5, 160.1, 152.0, and 145.9 mAh g^−1^ at 0.1, 0.2, 0.5, and 1 C (1st cycle of each C-rate), respectively (Figure 4b). Upon returning to 0.1 C, the GPE showed a discharge capacity of 160.3 mAh g^−1^, recovering 96.9% of the initial capacity.

In contrast, the developed PIL-GPE (8:2) demonstrated enhanced capacities of 198.8, 194.3, 185.3, and 176.6 mAh g^−1^ at 0.1, 0.2, 0.5, and 1 C, respectively. Upon returning to 0.1 C, the PIL-GPE (8:2) showed a discharge capacity of 188.2 mAh g^−1^, recovering 94.7% of the initial discharge capacity (Figure 4c). Figure 4d compares the capacity utilization ratios of GPE and PIL-GPE (8:2) at varying C-rates. The GPE had 96.7%, 91.8%, and 88.1% capacity retention values at 0.2, 0.5, and 1 C, respectively, whereas the PIL-GPE (8:2) showed higher capacity retention, maintaining 97.8%, 93.2%, and 88.9% at 0.2, 0.5, and 1 C, respectively, to their 0.1 C capacities.

This improved discharge capacity and capacity retention of the PIL-GPE (8:2) compared to the GPE at all the C-rates can be due to the superior ionic conductivity and lithium transference number (*t_Li+_*) of the PIL-GPE (8:2), higher than the previously reported values. (Table S1). [37,38,39,40,41].

**Figure 5:**
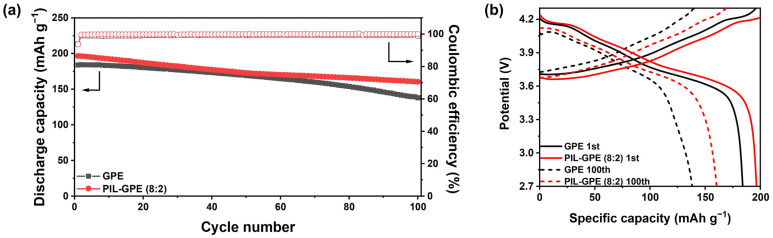
(**a**) Cycling performance of GPE and PIL-GPE (8:2) at 0.3 C; (**b**) charge/discharge profile of GPE and PIL-GPE (8:2) at 1st and 100th cycles.

The cycling life performance of the GPE and the PIL-GPE (8:2) was evaluated through repeated charge and discharge cycles at 0.3 C to assess the long-term stability. As shown in Figure 5a, the GPE demonstrated an initial discharge capacity of 184.1 mAh g^−1^ and became 138.2 mAh g^−1^ after 100 cycles, corresponding to 75.1% of its initial capacity. In contrast, the PIL-GPE (8:2) exhibited a higher initial discharge capacity of 196.6 mAh g^−1^ and superior cycling stability, maintaining a capacity of 160.4 mAh g^−1^ after 100 cycles, which corresponds to 81.6% retention (Figure 5b).

Furthermore, the PIL-GPE (8:2) achieved an initial coulombic efficiency of 93.6% and an average coulombic efficiency of 99.8%, demonstrating improved charge–discharge reversibility compared to the GPE, which exhibited an initial coulombic efficiency of 99.6% and an average coulombic efficiency of 99.3%. These findings suggest that in situ polymerization via E-beam irradiation without initiators offers excellent processability and enhances electrochemical properties and cell performance when polymer networks incorporate polymerizable ionic liquids. This approach demonstrates strong potential for improving the lifespan and efficiency of lithium-ion batteries.

## 3. Conclusions

In this work, we developed and demonstrated, for the first time, a gel polymer electrolyte (GPE) based on a polymerizable ionic liquid (PIL) using high-energy E-beam irradiation, which effectively penetrates metal casing and eliminates the need for initiators. Incorporating PIL into the polymer network improved its electrochemical properties, including the ionic conductivity, the electrochemical stability, and the lithium transference number, due to the electrostatic interaction between the imidazolium-based cationic group of the PIL and the Li^+^ and TFSI^−^ ions. Furthermore, the PIL-GPE (8:2) demonstrated superior thermal stability compared to the GPE made with ETPTA alone in the polymer network.

The coin cell with the NCM811 cathode using the PIL-GPE (8:2) achieved a discharge capacity of ~200 mAh g^−1^ and exhibited more stable cycling performance than the GPE without PIL. These results highlight the potential for high-performance lithium-ion batteries (LIBs) based on the GPEs processed via E-beam irradiation, offering excellent processability and improved electrochemical stability.

## 4. Materials and Methods

### 4.1. Materials

Lithium bis(trifluoromethanesulfonyl)imide (LITFSI, ≥99.95%, Merck Co., Rahway, NJ, USA), trimethylolpropane ethoxylate triacrylate (ETPTA, M_n_ ~428, Merck Co., Rahway, NJ, USA), fluoroethylene carbonate (FEC, >98%, TCI Chemical, Tokyo, Japan), 2-bromoehtyl acrylate (94%, Thermo Fisher Scientific, Waltham, MA, USA), 1-butylimidazole (99%, Alfa Aesar, Heysham, UK), Dichloromethane (DCM, Merck Co., Rahway, NJ, USA), acetonitile (MeCN, Merck Co., Rahway, NJ, USA), N-methyl-2-pyrrolidone (NMP, Merck Co., Rahway, NJ, USA), and succinonitrile (SN, Merck Co., Rahway, NJ, USA) were used without further purifications.

### 4.2. Synthesis of Ionic Liquid

**Figure 6:**
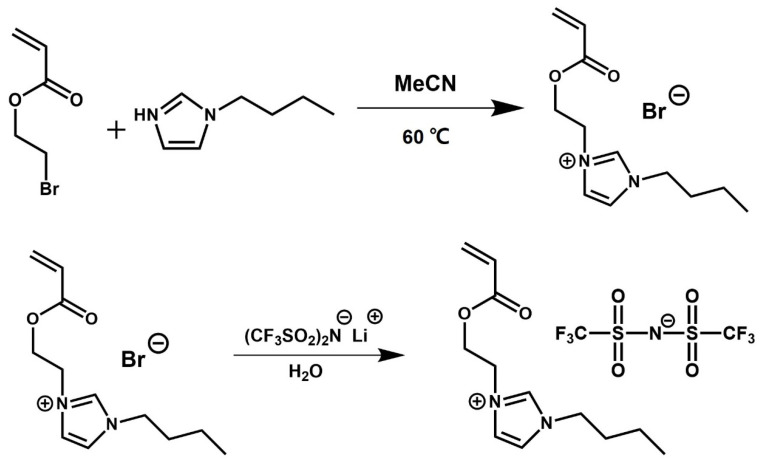
Synthetic scheme of [AEBI][TFSI] ionic liquid.

The synthetic scheme of the 1-[2-acryloyloxyethyl]-3-buthylimidazolium bis(trifluoromethanesulfonyl)imide ([AEBI][TFSI]) monomer is shown in Figure 6. The synthesis was carried out following a previously reported method [28]. Under a high purity nitrogen atmosphere, 5 g (27.9 mmol) of 2-bromoehtyl acrylate and 3.64 g (29.31 mmol, 1.05 eq.) of 1-butylimidazole were mixed in 30 mL of MeCN and stirred at 60 °C overnight. After the end of the reaction, MeCN was removed using a rotary evaporator. 1–[2–acryloyloxyethyl]–3–buthylimidazolium bromide ([AEBI]Br) was extracted by DI water followed by washing with DCM at least three times. For the anion exchange, 8.41 g (29.295 mmol, 1.05 eq.) of LiTFSI was added to an aqueous solution of [AEBI]Br and stirred overnight at room temperature. After the anion exchange, [AEBI][TFSI] was extracted using DCM, followed by washing with DI water and repeated with AgNO_3_ until no residue was detected. The product was dried in a vacuum oven to remove residual moisture. The ^1^H-NMR and (b) ^19^F-NMR spectra of [AEBI][TFSI] product are shown in Figure S2 (Supplementary Materials).

### 4.3. Preparation of Precursor

The plastic crystal electrolyte (PCE) was prepared by mixing 1 M LiTFSI powder with succinonitrile (SN) and stirring the mixture overnight, followed by the addition of fluoroethylene carbonate (FEC) at 5 vol% ratio to SN. A polymer network solution was then prepared using [AEBI][TFSI] and ETPTA in molar ratios of 7:3, 8:2, and 0:10 (PIL), respectively. To prepare the final GPE precursor, PCE and the polymer network solution were combined at a ratio of 85:15 (*w*/*w*). All the precursor solutions were prepared in an argon-filled glove box to ensure an inert environment.

### 4.4. Cell Fabrication

The cathode slurry was prepared by mixing N-methyl-2-pyrrolidone (NMP) with LiNi_0.8_Co_0.1_Mn_0.1_O_2_ (NCM 811, MTI Korea, Seoul, Republic of Korea), carbon black (CB, MTI Korea, Seoul, Republic of Korea), and polyvinylidene fluoride (PVDF, Solef 5130, Solvay S. A., Brussels, Belgium) in an 80:10:10 weight ratio using a swing planetary mixer (HSPM, Gunpo, Gyeonggi-do, HANTECH). The cathode slurry was then coated onto aluminum foil and vacuum dried at 100 °C for 8 h. The mass loading of the fabricated electrodes was 2–2.5 mg cm^−2^.

To assemble the CR2032-type coin cell, the previously prepared GPE precursor was injected onto the NCM 811 electrode, followed by the placement of glass fiber for mechanical support, a gasket, Li metal, a spacer, a spring, and a cap, in that order. The assembled coin cell was irradiated with an electron beam (GEV Co. Ltd., Eumseong, Chungcheongbuk-do, Republic of Korea) in 5 kGy increments from 10 to 30 kGy. The beam energy was set to 10 MeV, with a current of 2 mA and a conveyor speed of 6.89 m min^−1^, requiring approximately 4.4 s per 5 kGy of irradiation.

### 4.5. Characterization of Gel Polymer Electrolytes

Attenuated total reflection Fourier-transform infrared spectroscopy (ATR FT-IR, Spectrum3, Perkin Elmer, Shelton, CT, USA) measurements were performed in the wavenumber range of 650–4000 cm^−1^ to analyze the formation of the polymer network. To evaluate the thermal behavior of the GPEs, thermogravimetric analysis (TGA, TG 209 F3 Tarsus, NETZCH, Selb, Bavaria, Germany) was conducted at a heating rate of 20 °C min^−1^ in an N2 atmosphere from 30 to 700 °C. The gel fraction was determined by drying the GPEs for 48 h and calculated using the following equation:

$$Gel\;fraction=\frac{W_{d}}{W_{0}}\times 100\;(\%)$$

where *W*_0_ and *W_d_* are the weight of the GPEs before and after drying, respectively.

### 4.6. Electrochemical Measurements

The ionic conductivity of the GPEs was measured via electrochemical impedance analysis (EIS, VersaSTAT3, AMTEK, Suwon, Gyeonggi-do, Republic of Korea) at a 10 mV amplitude from 10^6^ Hz to 10^0^ Hz and was calculated using the equation below

$$\sigma =\frac{t}{\left(A\times R_{b}\right)}$$

where *A* is the area of the stainless-steel electrode, *R_b_* is the bulk resistance of the polymer electrolyte, and *t* is thickness of the polymer electrolyte. Linear sweep voltammetry (LSV) was carried out using the same electrochemical analyzer (VersaSTAT3) with a stainless-steel/electrolytes/Li metal asymmetric cell versus Li/Li^+^ in the voltage range of 0.0 to 6.0 V at a scan rate of 1 mV s^−1^ for measuring the oxidation stability of the GPEs.

The lithium transference number (*t_Li+_*) of the GPEs was carried out via chronoamperometry and EIS measurements with a Li metal/electrolytes/Li metal symmetric cell, and calculated using the equation below

$$t_{Li+}=\frac{l_{s}\left(∆V-l_{o}R_{o}\right)}{l_{o}\left(∆V-l_{s}R_{s}\right)}$$

where *l_o_* and *l_s_* are initial and steady-state DC current values, respectively, and *R_o_* and *R_s_* are the interfacial resistance before and after DC polarization, respectively. Δ*V* (10 mV) is the applied voltage.

## Figures and Tables

**Table 1:** Discharge capacity of GPEs at different discharge rates.

GPEs	Discharge Capacity (mAh g^−1^)				
	0.1 C	0.2 C	0.5 C	1 C	0.1 C
GPE	165.5	160.1	152.0	145.9	160.3
PIL-GPE (8:2)	198.8	194.3	185.3	176.6	188.2

## Data Availability

The data presented in this study are available on request from the corresponding author.

## Supplementary Materials

The following supporting information can be downloaded at https://www.mdpi.com/article/10.3390/gels10120798/s1, Figure S1: Polymerization scheme of PIL-GPEs; Figure S2: (a) ^1^H-NMR and (b) ^19^F-NMR spectra of [AEBI][TFSI]; Table S1: Summary of NCM811/GPE/Li cell performance. [37,38,39,40,41].
