# Supplementary material for: Polymerizable Ionic Liquid-Based Gel Polymer Electrolytes Enabled by High-Energy Electron Beam for High-Performance Lithium-Ion Batteries
Source: Gels. 2024 Dec 6;10(12):798. doi: 10.3390/gels10120798 (PMC11675922; doi:10.3390/gels10120798)
Supplement: Supplementary file 1 [file gels-10-00798-s001.zip › gels-3335241-supplementary.pdf]

Supplementary Materials

Polymerizable Ionic Liquid-Based Gel Polymer Electrolytes Enabled by High-Energy Electron Beam for High-Performance Lithium-Ion Batteries

Wookil Chae and Taeshik Earmme*

Department of Chemical Engineering, Hongik University, Seoul 04066, Republic of Korea
* Correspondence: earmme@hongik.ac.kr

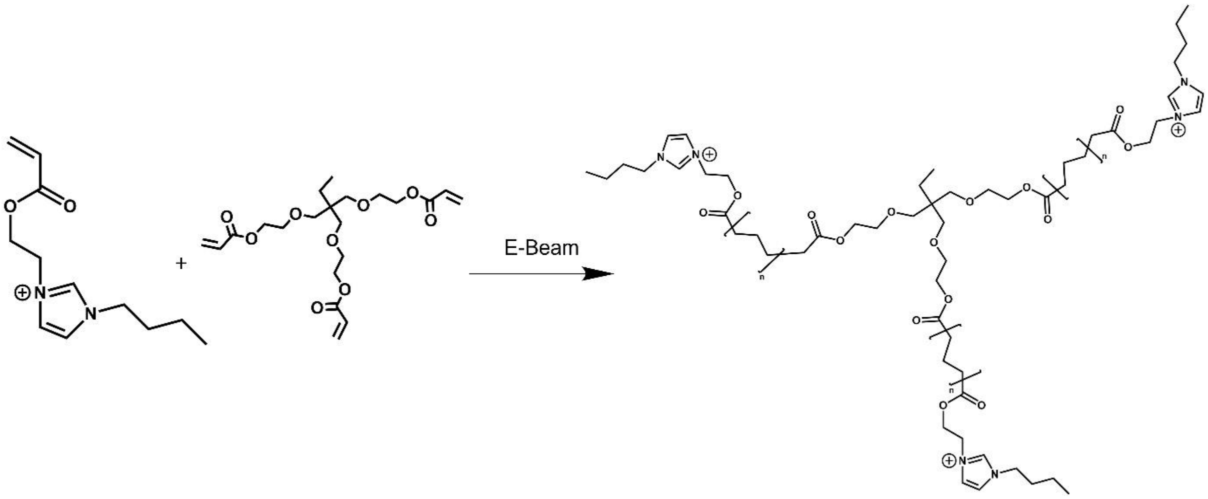

Figure S1. Polymerization scheme of PIL-GPEs

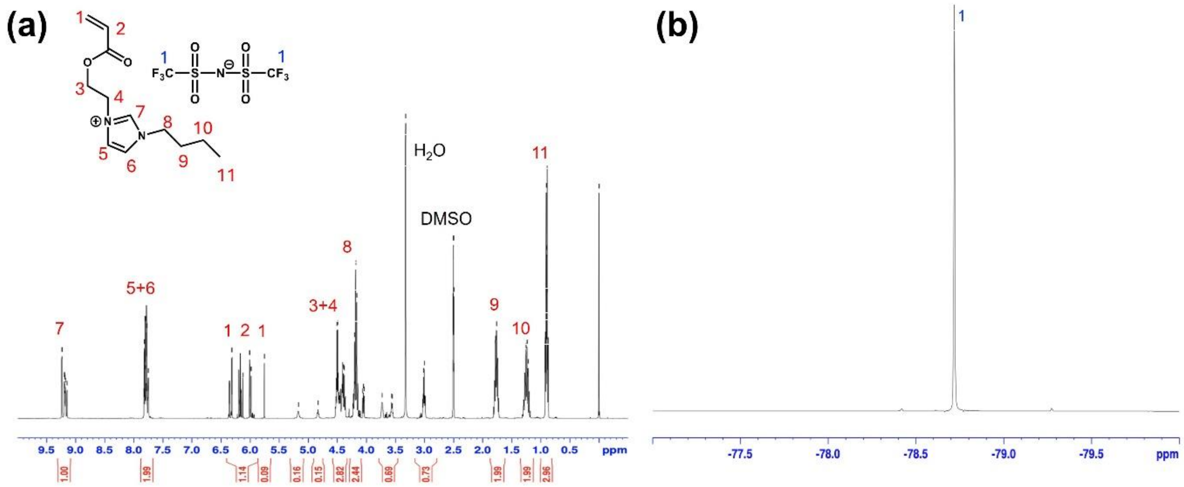

Figure S2. (a) ^{1}H-NMR and (b) ^{19}F-NMR spectra of [AEBI][TFSI]

Table S1. Summary of NCM811/GPE/Li cell performance.

Configuration	C-rate (C)	Discharge capacity (mAh g^{-1})	Temperature ()	Upper voltage (V)	Reference
NCM 811/PIL-GPE (8:2)/Li	0.1	198.8	25	4.3	This work
NCM 811/MTGPE/Li	0.1	198.8	25	4.3	[37]
NCM 811/PEGGPE@HT)/Li	0.2	194.2		4.3	[38]
NCM 811/PGPE/Li	0.1	190	30	4.3	[39]
NCM 811/quasi-solid electrolyte/Li	0.1	224.7	30	4.5	[40]
NCM 811/FGPE/Li	01	224.1	25	4.5	[41]
